# Genetic and Non-Genetic Variation of Milk Total Antioxidant Activity Predicted from Mid-Infrared Spectra in Holstein Cows

**DOI:** 10.3390/ani10122372

**Published:** 2020-12-10

**Authors:** Giovanni Niero, Angela Costa, Marco Franzoi, Giulio Visentin, Martino Cassandro, Massimo De Marchi, Mauro Penasa

**Affiliations:** 1 Department of Agronomy, Food, Natural Resources, Animals and Environment (DAFNAE), University of Padova, Viale dell’Università 16, 35020 Padova, Italy; g.niero@unipd.it (G.N.); marco.franzoi89@gmail.com (M.F.); martino.cassandro@unipd.it (M.C.); massimo.demarchi@unipd.it (M.D.M.); mauro.penasa@unipd.it (M.P.); 2 Department of Veterinary Medical Sciences (DIMEVET), Alma Mater Studiorum—University of Bologna, Via Tolara di Sopra 50, 40064 Bologna, Italy; giulio.visentin@unibo.it

**Keywords:** dairy, antioxidant, casein, milk fat, genetic correlation, proxy

## Abstract

**Simple Summary:**

The total antioxidant activity (TAA) of food is important for human health and results from the contribution of different nutraceutical compounds. Direct determination of TAA in food is time-consuming and expensive. Infrared technologies allow the prediction of difficult-to-measure traits with certain accuracy in several organic matrices, including TAA of bovine milk. In order to understand the background of TAA and identify potential strategies to improve this feature in bovine milk, we explored its non-genetic sources of variation and estimated heritability and correlations with traits of economic interest in a large database of Holstein cows.

**Abstract:**

Food antioxidants enhance products shelf life and stability during technological treatments through the maintenance of their physical and chemical properties. Moreover, they are endowed with several positive effects on human health, including cell membranes preservation, enzyme functionality, and DNA integrity. Milk has been described in relation to a wide array of fat soluble and water-soluble antioxidant compounds, in particular vitamin A, C, and E, lactoferrin and peptides derived from casein and whey proteins. The total antioxidant activity (TAA) of milk is a novel and scarcely explored trait, defined as the sum of antioxidant contributions of the aforementioned compounds. On this background, the aims of the present study were to investigate the variability of milk TAA on a large scale exploiting predictions obtained through mid-infrared (MIR) spectroscopy and to estimate genetic parameters of this trait in Holstein cows. Individual milk samples were collected between January 2011 and December 2018 during the routine milk recording procedure. Samples were analysed for gross composition through MIR spectroscopy and MIR spectra were stored. Milk TAA was then predicted (pTAA) from the stored milk MIR spectra (111,653 test-day records of 9519 cows in 344 herds) using the previously developed prediction model; considering the prediction accuracy, pTAA might be considered a proxy of the TAA determined through the reference method. Overall, pTAA averaged 7.16 mmoL/L of Trolox equivalents, showed a nadir around 40 days after calving and increased thereafter, following a linear trend up to the end of lactation. The lowest pTAA was observed in milk sampled from June to September. Milk pTAA was heritable (0.401 ± 0.015) and genetically associated to fat yield (0.366 ± 0.049), crude protein (CP) yield (0.238 ± 0.052), fat percentage (0.616 ± 0.022) and CP percentage (0.754 ± 0.015). The official selection index of Italian Holstein put the 49% of the emphasis on fat and protein yield and percentage; therefore, it derives that an indirect favourable selection for milk pTAA should be already in progress in Italian Holstein population.

## 1. Introduction

Milk and dairy antioxidants have been attracting the attention of scientific community for their technological implications in the food industry and their importance for human health through the daily diet. Antioxidants are involved in the maintenance of physical properties and chemical composition of dairy products, especially during milk technological treatments (e.g., pasteurization), cheese ripening, and milk and cheese shelf life [1], and they exert a central role in the prevention of milk off-flavours, by protecting lipids from auto-oxidation [2]. Moreover, dietary antioxidants have been described for their activity towards the neutralisation of free radicals and reactive oxygen species, and thus for their positive effects on human health [3]. Reactive oxygen species lead to several injuries at cytological and molecular levels, with particular regard to cell membrane lipid peroxidation, DNA cleavage, alteration of protein folding, and enzymes inactivation [4]. Among the negative effects of oxidative stress, the increased risk of clinical diseases stands out, particularly atherosclerosis, rheumatoid arthritis, diabetes, and some forms of cancer.

Milk antioxidants include a wide array of free radical scavenging molecules. In this respect, conjugated linoleic acids have been described empirically as one of the most bioactive antioxidant compounds in milk fat, even if the biochemical mechanisms responsible for this physiological effect remain uncertain [5]. Moreover, milk fat globules contain several antioxidant vitamins, mainly vitamin A (retinol), vitamin A precursor (β-carotene), and vitamin E (tocopherols) [5]. The most important hydrophilic antioxidants in milk include vitamin C (ascorbate) [6], low molecular weight thiols [7], whey proteins (particularly lactoferrin), and peptides derived from whey protein hydrolysis or fermentation [8]. Caseins have also shown antioxidant activity; in particular, the effectiveness of caseins as antioxidant compounds has been associated to the specific composition in amino acids and thus to different genetic variants [9], cheese ripening progression [10], and hydrolysis and fermentation rates [8].

Milk total antioxidant activity (TAA) has been defined as the sum of antioxidant contribution of the aforementioned molecules and compounds [11]. Milk TAA can be measured in a colorimetric reaction monitored through a spectrophotometric assay and is quantified as Trolox equivalents (TEs), where Trolox ([±]-6-hydroxy-2,5,7,8-tetra-methylchoromane-2-carboxylic acid) is a synthetic antioxidant used in laboratory for analytical purposes [11]. Sources of phenotypic variation for milk TAA have been recently investigated by Niero el al. [12], who measured TAA through the spectrophotometric reference method in milk of Holstein (HO) cows. The same authors assessed the ability of mid-infrared (MIR) spectroscopy to predict this novel phenotype.

To our knowledge, no studies have investigated the phenotypic and genetic aspects of milk TAA at population level because phenotyping through reference analysis is costly and time-consuming. Predictions obtained from milk spectra can be feasibly exploited, since MIR-predicted TAA (pTAA) can be considered a proxy of the real milk TAA. The aims of the present study were to (i) investigate non-genetic factors of pTAA and (ii) estimate its genetic parameters and correlations with milk yield traits, composition, somatic cell score (SCS), and detailed protein fractions in a large database of Italian HO cows.

## 2. Materials and Methods 

### 2.1. Data

A total of 473,816 milk samples of 23,450 HO cows were collected between January 2011 and December 2018 during the monthly official milk recording scheme. Test-day milk yield (kg/day), days in milk (DIM), parity, and herd of animals were provided by the Breeders Association of Bolzano Province (Bolzano, Italy). Immediately after collection, milk samples (50 mL) were added with 200 μL of preservative (Bronysolv; ANA.LI.TIK Austria, Vienna, Austria) and analysed in the laboratory of the South Tyrolean Dairy Association (Bolzano, Italy) for fat, crude protein (CP), casein (CN), and lactose percentages using MilkoScan FT6000 or MilkoScan FT7 (FOSS, Hillerød, Denmark). To ensure the comparability of spectra, MilkoScan FT6000 and MilkoScan FT7 were routinely calibrated using standard samples, as recommended by manufacturer instructions. Moreover, a principal component analysis on spectra was performed and did not show significant differences between the 2 instruments. Test-day fat and CP yields (kg/day) were calculated from milk yield and fat and CP percentages, and milk somatic cell count (SCC, cells/μL) was determined through Fossomatic 5500 (FOSS, Hillerød, Denmark) and transformed to SCS using the formula SCS = 3 + log_2_(SCC/100).

For each milk sample, spectral information containing 1060 infrared transmittance data in the region between 5000 and 900 cm^−1^ were stored and used for a posteriori prediction of milk detailed protein composition (% of CP) and TAA (mmoL/L TE), since these phenotypes are not routinely determined in milk laboratories during official milk testing procedures. Detailed protein composition was predicted from milk MIR spectra using prediction models developed by Niero et al. [13]; in particular, the coefficients of determination of the models in cross validation were 0.88, 0.60, 0.74, 0.37, and 0.47, and ratios of performance to deviation were 2.86, 1.60, 2.03, 1.30, and 1.34 for α-casein, β-casein, κ-casein, α-lactalbumin, and β-lactoglobulin, respectively. Regarding milk TAA, this new phenotype was predicted from milk MIR spectra using the prediction model developed by Niero et al. [12] and thus full details on samples collection, analytical reference method, and the procedure to develop the model for TAA can be retrieved from those authors. Briefly, 1249 individual milk samples of HO cows were analysed for TAA through the reference spectrophotometric method and the prediction model was developed on the same dataset using partial least squares regression analysis. The coefficient of determination in cross validation was 0.46 and the ratio of performance to deviation was 1.30. According to the most recent literature [14,15], models with similar fitting statistics have been reported for other traits and have been considered enough accurate for screening and genetic purposes on a large scale. The Mahalanobis distance between the data point (spectrum) and the centroid of spectra included in the calibration set was used to identify and remove spectral outliers from the dataset. Finally, pTAA values outside the range of the reference data used for calibrations [12] were discarded from the dataset.

### 2.2. Editing and Statistical Analysis

Lactations outside the range 5 to 305 DIM and with less than 5 test-day records were discarded from the dataset. Contemporary groups were defined as cows sampled in the same herd-test-date (HTD), and only HTD with at least 5 cows were retained. Cows that changed herd during the investigated period and those with unknown parents were removed from the data. Finally, values of milk pTAA, yield and composition traits, SCS, and protein fractions that deviated more than 3 standard deviations from the respective mean were treated as missing. The final dataset included 111 653 records of 9519 cows in 344 herds.

A linear model was imputed in ASReml v4.1 [16] to estimate least squares means of fixed effects and genetic parameters; in particular, variance and covariance components were obtained through univariate and bivariate analyses, respectively:

y*_ijklmn_* = µ + Parity*_i_* + DIM*_j_* + HTD*_k_* + Cow*_l_* + Animal*_m_* + e*_ijklmn_* (1)

where y*_ijklmn_* is the investigated trait; µ is the overall intercept of the model; Parity*_i_*is the fixed effect of the *i*th parity of the cow (*i* = 1 to 5, with class 5 including parities ≤ 14); DIM*_j_*is the fixed effect of the *j*th class of DIM of the cow (*j* = 1 to 30, each class being 10 d wide); HTD*_k_*is the fixed effect of the *k*th contemporary group (*k* = 1 to 10 504); Cow*_l_* is the random permanent environmental effect of the *l*th cow (*l* = 1 to 9519) ~N(0, I$\sigma_{w}^{2}$), where I is an identity matrix of appropriate order and $\sigma_{w}^{2}$ is the permanent environmental variance; Animal*_m_* is the random additive genetic effect of the *m*th animal ~N(0, A$\sigma_{a}^{2}$), where A is the additive genetic relationship matrix and $\sigma_{a}^{2}$ is the additive genetic variance; and e*_ijklmn_* is the random residual effect ~N(0, I$\sigma_{e}^{2}$), where $\sigma_{e}^{2}$ is the residual variance. The matrix A included cows with phenotypic information (*n* = 9519) and six generations of ancestors (i.e., 31,645 animals in total). A multiple comparison of least squares means of pTAA for the fixed effects was performed using Bonferroni’s post-hoc test (*p* < 0.05).

The phenotypic variance ($\sigma_{p}^{2}$) was derived by summing up $\sigma_{w}^{2}$, $\sigma_{a}^{2},$ and $\sigma_{e}^{2}$. Heritability (h^2^), repeatability (t), and phenotypic (r_p_) and genetic correlations (r_a_) were calculated from variance and covariance components as:

(2) $$h^{2}=\frac{\sigma_{a}^{2}}{\sigma_{a}^{2}\;+\;\sigma_{w}^{2}\;+\;\sigma_{e}^{2}},\;t=\frac{\sigma_{a}^{2}\;+\;\sigma_{w}^{2}}{\sigma_{a}^{2}\;+\;\sigma_{w}^{2}\;+\;\sigma_{e}^{2}},\;r_{p}=\frac{\sigma_{p12}}{\sqrt{\sigma_{p1}^{2}*\;\sigma_{p2}^{2}}},\;\mathrm{and}\;r_{a}=\frac{\sigma_{a12}}{\sqrt{\sigma_{a1}^{2}*\;\sigma_{a2}^{2}}},$$

where $\sigma_{p12}$ and $\sigma_{a12}$ are the phenotypic and the additive genetic covariances between trait 1 and trait 2; $\sigma_{p1}^{2}$ and $\sigma_{p2}^{2}$ are the phenotypic variances of traits 1 and 2; and $\sigma_{a1}^{2}$ and $\sigma_{a2}^{2}$ are the additive genetic variances of traits 1 and 2. To estimate least squares means of calendar month and year of sampling, a supplementary analysis was performed with the same model but excluding the effect of HTD and adding month and year of sampling as fixed effects. Finally, Pearson correlations between sires’ estimated breeding value (EBV) of the traits with accuracy ≥0.65 were assessed.

## 3. Results and Discussion

Overall, MIRS equations used to predict milk TAA and detailed protein composition are characterised by moderate to low accuracies, with particular regard to the coefficient of determination in cross validation. Such fitting statistics suggest that prediction models are not adequate for analytical purposes (e.g., precise determination of TAA on a single milk sample or even on a batch of milk samples). Still, moderate to low accuracies are considered adequate for screening purposes at population level, and to estimate genetic parameters on repeated observations within single animal. Both these aspects are also known to be associated with a reduction of standard error of the predicted trait [14,15]. Literature shows that moderately accurate MIRS predictions can be successfully used to generate genetic parameters and derive EBV that are highly correlated to the EBV of the actual (measured) phenotype [17]. 

### 3.1. Descriptive Statistics

Milk pTAA averaged 7.16 mmol/L TE and ranged from 5.46 to 8.76 mmol/L TE (Table 1). The average pTAA was maximum and minimum in second- (7.19 mmol/L TE) and first-parity cows (7.13 mmol/L TE), respectively. These values are consistent with results reported by Niero et al. [18], who measured TAA on different types of commercial milk, and slightly greater than values obtained by Niero et al. [12], who measured TAA using the reference spectrophotometric method on 1249 milk samples of HO cows. 

The average pTAA of the present study was faintly greater than TAA measured in goat (6.80 mmol/L TE) and lower than TAA measured in buffalo (7.35 mmol/L TE) and sheep milk (7.78 mmol/L TE) [19]. It is likely that TAA mirrors the specific milk composition of these dairy species, with specific regard to fat, protein, and CN percentage. In fact, the relatively low TAA of cow and goat milk could be associated to a lower fat, protein, and CN percentage; on the other hand, high TAA (buffalo and sheep milk) is correlated with greater milk solids content. The coefficient of variation of pTAA (7.51%) was lower than that of fat, CP, and CN percentage, and greater than that of lactose percentage (Table 1). In general, coefficients of variation observed in the present study were lower than those obtained by Niero et al. [12], likely due to the different sample size and, in the case of TAA, to the methods to determine this trait.

### 3.2. Non-Genetic Factors Affecting Milk TAA

**Figure 1:**
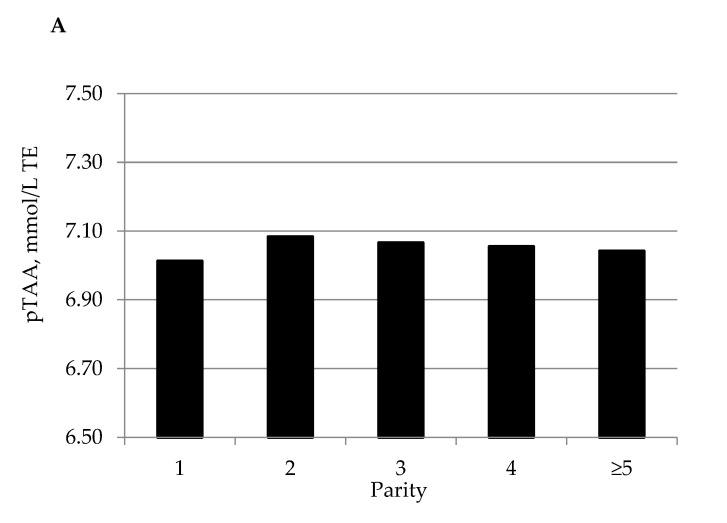

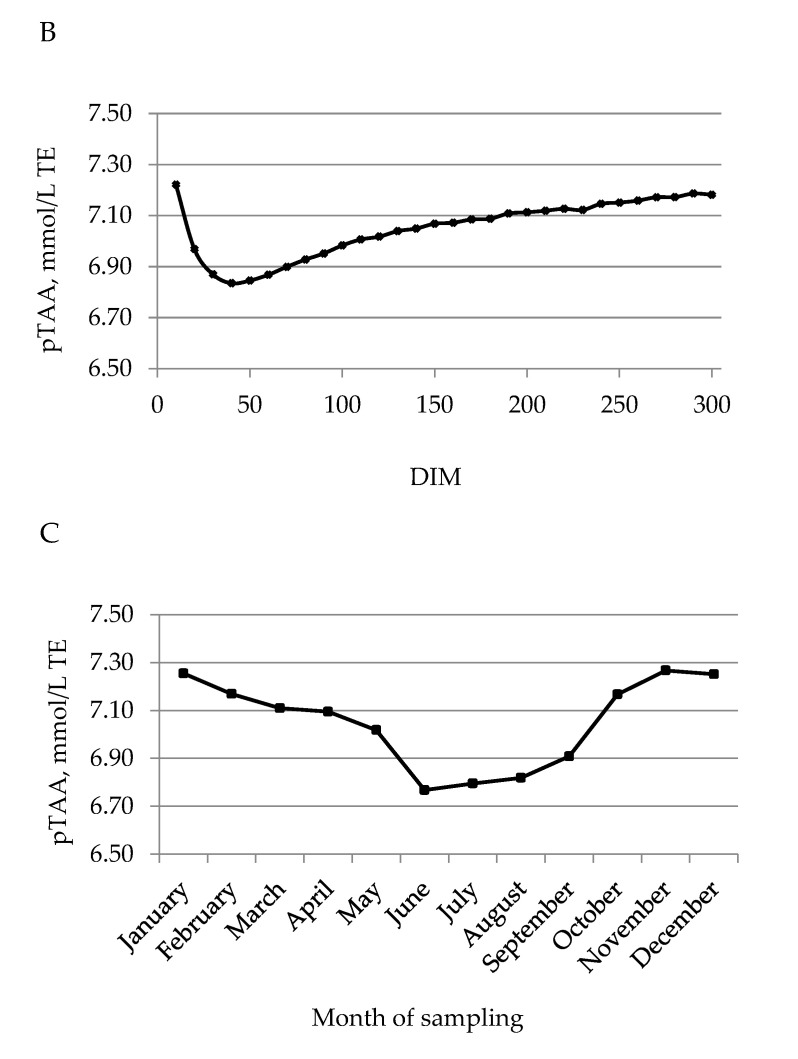
Least squares means of bovine milk predicted total antioxidant activity (pTAA) expressed as mmol/L of Trolox equivalents (TEs) for the fixed effects of (**A**) parity (SE < 0.008), (**B**) days in milk (DIM; SE < 0.009), and (**C**) month of sampling (SE < 0.015).

All the fixed effects included in the statistical model (i.e., parity, DIM, and calendar month and year of sampling) were significant in explaining the variation of milk pTAA. The pTAA differed (*p* < 0.001) between first- (7.01 mmol/L TE) and second-parity cows (7.08 mmol/L TE; Figure 1). In general, pTAA slightly decreased from second parity onward. The same trend was observed by Franzoi et al. [20] for CP percentage, as well as for α-casein, β-casein, κ-casein, and α-lactalbumin. This comparison supports the hypothesis that protein, CN, and whey proteins contribute to milk TAA [8,9,10]. Milk pTAA decreased from 7.22 to 6.83 mmol/L TE when moving from 10 to 40 DIM and increased thereafter up to 7.18 mmol/L TE at the end of lactation (Figure 1). This trend is opposite to that of milk yield, leaving room for the hypothesis of a dilution effect. In fact, the lactation curve of milk pTAA (Figure 1) resembled that of CP percentage and its fractions [20] and fat percentage [21]. Considering the effect of month of sampling, milk collected in summer had lower pTAA than milk sampled in other seasons. In particular, the lowest pTAA was obtained for milk collected in June (6.77 mmol/L TE) and the greatest for milk collected in November (7.25 mmol/L TE), December and January (7.27 mmol/L TE). The impact of month of sampling on milk pTAA could be the result of management practices throughout the year, like the practicing of extensive pasture from late spring to the end of summer, that has been associated to an overall reduction of protein [20] and fat [21] content in bovine milk.

### 3.3. Genetic Parameters of Milk TAA

Heritability of pTAA (0.401 ± 0.015; Table 2) was intermediate between h^2^ of fat percentage (0.358 ± 0.015) and CP percentage (0.472 ± 0.017). This was somehow expected, since TAA is the direct combination of the antioxidant actions of several milk compounds, mainly contained in fat and protein, and it is reasonable to assume that it is predicted from the same spectral regions of fat and protein [12]. To our knowledge, this is the first study that estimated genetic parameters of pTAA in bovine milk and thus the comparison with the literature was not possible. Nevertheless, h^2^ of pTAA was in line with h^2^ of fat and protein percentage for HO cattle worldwide [22], particularly in Italy [23,24], Denmark [25], and Canada [26]. Similarly, the repeatability of TAA (Table 2) was between that of fat percentage (0.468 ± 0.006) and CP percentage (0.622 ± 0.005). Despite the moderate h^2^, the coefficient of genetic variation of pTAA was low (2.42%); this would make direct selection for this trait challenging in dairy cattle, as it has been reported for lactose percentage [23,24], a trait with coefficient of genetic variation similar to pTAA.

### 3.4. Correlations

The negative association between milk yield and pTAA at phenotypic level (r_p_ = −0.184 ± 0.007) highlighted that high producing cows were characterised by relatively lower milk TAA than less producing animals, supporting the previously discussed hypothesis of a dilution effect. In addition, there was a negative r_a_ between the two traits, which indicates that genetic selection focused only on milk yield would be detrimental for milk pTAA in the long term (Table 3).

As regards milk gross composition, the strongest correlations were assessed between pTAA and CP percentage (r_p_ = 0.610 ± 0.005 and r_a_ = 0.754 ± 0.015), and TAA and CN percentage (r_p_ = 0.589 ± 0.005 and r_a_ = 0.733 ± 0.016), whereas pTAA and lactose percentage were unrelated (Table 3). Lactose is the main sugar in mammals’ milk and the most abundant solid in bovine milk [23]; although it contributes to milk nutritional value, higher lactose percentage does not translate into a greater milk TAA for chemical and biological reasons [23]. Fat percentage was moderately associated with MIR-predicted TAA, both phenotypically (r_p_ = 0.407 ± 0.006) and genetically (r_a_ = 0.616 ± 0.005). These favourable relationships were somewhat expected since milk fat contains a relevant number of antioxidant compounds such as molecules belonging to vitamin A and vitamin E families [27]. Thus, it is likely that an increase of milk fat percentage would result in an increase of fat-soluble antioxidant content [28]. In particular, a simultaneous increase of milk conjugated linoleic acids is expected to increase milk TAA. The r_p_ between pTAA and fat percentage was considerably greater than the estimate (0.13) reported by Niero et al. [12], who calculated Pearson correlations using milk TAA measured with the reference method. All CN fractions were positively, despite weakly, related to pTAA; in particular, the strongest genetic and phenotypic associations were estimated with α-CN and β-CN, respectively. The lack of association of pTAA with α-lactalbumin and β-lactoglobulin (Table 3) indicated that changes in milk whey proteins do not affect pTAA and that caseins are mostly responsible for the protein antioxidant effect.

Weak relationships were estimated between pTAA and SCS (r_p_ = 0.086 ± 0.006 and r_a_ = 0.109 ± 0.057), meaning that, on average, antioxidant compounds are more abundant in milk with greater SCS. Atakisi et al. [29] observed that subclinical mastitis causes oxidative alteration of cow milk and increases the production of nitric oxide radical species. The strong development of free radicals may result in amplified antioxidant response by mammary gland cells [30,31], which explains the positive relationship between pTAA and SCS. Finally, it is worth considering that SCS is usually weakly positively related to fat and protein percentages in cattle at both genetic and phenotypic level [23,32,33]; therefore, positive, despite weak, relationships between SCS and TAA were somehow expected.

**Figure 2:**
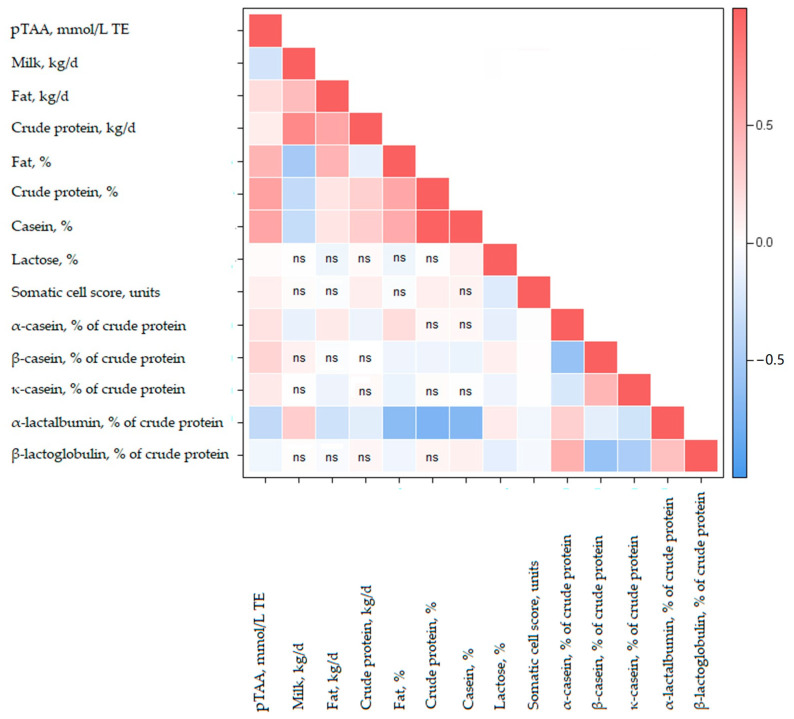
Pearson correlations (*p* < 0.001) between sires’ estimated breeding value (EBV; *n* = 442, accuracy ≥ 0.65) of bovine milk predicted total antioxidant activity (pTAA) expressed as mmol/L of Trolox equivalent (TE) and EBV of other traits. ns = not significant.

Pearson correlations between sires’ EBV of pTAA and EBV of other traits are depicted in Figure 2. Overall, the estimates mirrored the r_p_ and r_a_ (Table 3) and confirmed pTAA to be related to fat and CP, more as percentage than yield. Similarly to r_p_ and r_a_, the correlation between EBV for pTAA and EBV for milk yield was negative and moderately weak (Figure 2). Considering that the official selection index of the Italian HO gives a null weight to milk yield but emphasises protein and fat yields and percentages (overall, they account for 49% of the weight in the total merit index) [22], it can be reasonably assumed that pTAA is currently subjected to indirect favourable selection in this population. Further research on pTAA, as for other traits of interest for human health, could be also exploited to maximize milk and dairy products added value [34].

## 4. Conclusions

The present study investigated the non-genetic variation of MIR-predicted milk TAA and estimated its h^2^ and correlations with yield and quality traits of HO cows. Milk pTAA was maximum in second-parity cows, exhibited a pattern across lactation that resembled that of fat and CP percentages, and varied across months of sampling, with the lowest values in summer and the greatest in winter. Genetic analysis revealed that milk pTAA had heritability comparable to that of fat and CP percentages, but lower genetic variation. Correlations of pTAA with milk yield were negative, whereas those with fat and CP were positive. Considering the low genetic variation of pTAA and that the official selection index of Italian HO includes both yields and percentages of fat and protein, an indirect improvement of milk pTAA is in progress.

## Figures and Tables

**Table 1:** Mean, coefficient of variation (CV), minimum and maximum of bovine milk predicted total antioxidant activity (pTAA), yield and composition traits, somatic cell score, and protein fractions.

Trait	*n*	Mean	CV (%)	Minimum	Maximum
pTAA (mmol/L of Trolox Equivalent)	111,653	7.16	7.51	5.46	8.76
Yield (kg/day)					
Milk	111,653	30.05	24.19	5.70	52.70
Fat	110,754	1.17	24.89	0.26	2.10
Crude protein	111,331	0.98	21.98	0.33	1.63
Milk composition (%)					
Fat	111,653	3.95	15.20	1.76	6.16
Crude protein	111,650	3.29	9.87	2.18	4.40
Casein	111,649	2.59	9.72	1.72	3.47
Lactose	111,653	4.79	3.30	4.13	5.37
Somatic cell score (units)	111,653	2.55	72.61	−3.64	9.62
Protein fractions (% of crude protein)					
α-casein	111,043	44.24	7.23	26.84	59.63
β-casein	109,500	28.66	14.32	13.80	53.85
κ-casein	108,712	16.84	20.18	7.46	33.29
α-lactalbumin	111,137	2.32	11.17	1.42	3.50
β-lactoglobulin	106,896	8.84	35.51	1.42	24.42

**Table 2:** Additive genetic variance ($\sigma_{a}^{2}$), cow permanent environmental variance ($\sigma_{w}^{2}$), heritability (h^2^), and repeatability (t) of bovine milk total antioxidant activity.

Parameter	Estimate	SE
$\sigma_{a}^{\;2}$	0.030	0.001
$\sigma_{w}^{\;2}$	0.008	0.001
$h^{2}$	0.401	0.015
t	0.500	0.006

**Table 3:** Phenotypic (r_p_) and genetic (r_a_) correlations of bovine milk predicted total antioxidant activity with yield and composition traits, somatic cell score, and protein fractions. Standard errors are given in parentheses.

Trait	r_p_	r_a_
Yield (kg/day)		
Milk	−0.184 (0.007)	−0.381 (0.045)
Fat	0.129 (0.006)	0.366 (0.049)
Crude protein	0.092 (0.007)	0.238 (0.052)
Milk composition (%)		
Fat	0.407 (0.006)	0.616 (0.022)
Crude protein	0.610 (0.005)	0.754 (0.015)
Casein	0.589 (0.005)	0.733 (0.016)
Lactose	−0.039 (0.009)	0.040 (0.030)
Somatic cell score (units)	0.086 (0.006)	0.109 (0.057)
Protein fractions (% of crude protein)		
α-casein	0.232 (0.007)	0.191 (0.032)
β-casein	0.153 (0.007)	0.243 (0.016)
κ-casein	0.078 (0.007)	0.173 (0.016)
α-lactalbumin	0.000 (0.000)	0.000 (0.000)
β-lactoglobulin	0.006 (0.001)	0.000 (0.000)
